# Visual Scanning Training, Limb Activation Treatment, and Prism Adaptation for Rehabilitating Left Neglect: Who is the Winner?

**DOI:** 10.3389/fnhum.2013.00360

**Published:** 2013-07-08

**Authors:** Konstantinos Priftis, Laura Passarini, Cristina Pilosio, Francesca Meneghello, Marco Pitteri

**Affiliations:** ^1^Laboratory of Neuropsychology, IRCCS San Camillo Hospital, Lido-Venice, Italy; ^2^Department of General Psychology, University of Padova, Padova, Italy

**Keywords:** prism adaptation, limb activation treatment, visual scanning training, neglect, rehabilitation, stroke

## Abstract

We compared, for the first time, the overall and differential effects of three of the most widely used left neglect (LN) treatments: visual scanning training (VST), limb activation treatment (LAT), and prism adaptation (PA). Thirty-three LN patients were assigned in quasi-random order to the three groups (VST, LAT, or PA). Each patient received only one type of treatment. LN patients’ performance on everyday life tasks was assessed four times (over a period of 6 weeks): A1 and A2 (i.e., the two pre-treatment assessments); A3 and A4 (i.e., the two post-treatment assessments). LN patients in each of the three treatment conditions were treated for the same number of sessions (i.e., 20). The results showed that improvements were present in the majority of the tests assessing the peripersonal space in everyday life activities. Our findings were independent of unspecific factors and lasted for at least 2 weeks following the end of the treatments. There were no interactions, however, between LN treatments and assessments. We suggest that all three treatments can be considered as valid rehabilitation interventions for LN and could be employed for ameliorating LN signs.

## Introduction

Left neglect (LN) is one of the most frequent and disabling neuropsychological syndromes following right-hemisphere damage. LN patients fail to report, orient to, or verbally describe stimuli in the contralesional, left side of space (Karnath et al., 2002; Heilman et al., 2003). Although, to date, there is no comprehensive theoretical account of LN, most authors sustain that LN patients are not aware of events on the left side of space, because they do not orient their spatial attention leftward (for a brief review, see Priftis et al., 2011). Together with spatial attention deficits, LN is also associated with representational and non-spatial impairments (e.g., non-spatially lateralized sustained attention, spatial working memory, spatial remapping, etc.; for reviews, see Husain and Rorden, 2003; Pisella and Mattingley, 2004; Priftis et al., 2013). The lack of contralesional awareness in LN patients cannot be attributed to primary sensory or motor deficits. Indeed, double dissociations have been reported between LN and basic sensory-motor defects (Vallar, 1998). LN is not a unitary disorder because many LN subtypes have been described (for a taxonomy, see Vallar, 1998). For instance, LN may selectively impair the personal space (i.e., the space of the body), the peripersonal space (i.e., the reaching and grasping space), or the extrapersonal space (i.e., the locomotor space, beyond the reaching and grasping space; for review, see Vallar, 1998).

Functional recovery of LN patients can be severely affected (e.g., Paolucci et al., 1996; Kalra et al., 1997). Indeed, LN may considerably limit the overall effectiveness of rehabilitation interventions, often to a greater extent than more obvious motor, sensory, and speech deficits (Buxbaum et al., 2004). Although some spontaneous recovery occurs in the majority of LN patients after stroke, LN signs remain severe in many patients and may persist in the chronic phase (Stone et al., 1992; Jehkonen et al., 2000, 2007; Farnè et al., 2004; Nijboer et al., in press). Thus, LN is one of the major factors underlying poor functional outcome following stroke (Denes et al., 1982; Jehkonen et al., 2000; Buxbaum et al., 2004; Farnè et al., 2004).

Over the past 60 years, many different treatments for rehabilitating LN have been conceived and tested (for recent reviews, see Kerkhoff and Schenk, 2012; Riestra and Barrett, 2013). Early approaches to the treatment of LN were mainly based on the clinical experience of rehabilitation specialists, and they were less theory-driven than more recent approaches (Robertson, 1999). In contrast, in the last three decades a variety of different theory-driven LN-treatment techniques have been developed, on the basis of specific theories that aim to understand the underpinning mechanisms of LN (for review, see Robertson, 1999). Among other LN rehabilitation techniques, three of the most widely validated LN treatments are: visual scanning training (VST; Weinberg et al., 1977; Antonucci et al., 1995), limb activation treatment (LAT; Robertson and North, 1992), and prism adaptation treatment (PA; Rossetti et al., 1998; Frassinetti et al., 2002).

### Visual scanning training

Systematic VST programs, employing voluntary orienting of spatial attention toward the left side of space, have been developed in the last 40 years (e.g., Diller and Weinberg, 1977; Pizzamiglio et al., 1992; Antonucci et al., 1995). In these programs, which are inspired by behavior modification techniques, LN patients are trained to actively explore the contralesional side of space on different tasks (e.g., picture scanning, copying, reading, etc.). Their visual search can be systematically guided by contralesional cues (e.g., a visual stimulus of reference on the left) and by the examiner’s feedback. The difficulty and spatial extension of contralesional stimuli is progressively increased as a function of LN patients’ performance. Using this paradigm, significant improvements of LN signs, both in group studies and in single-case studies, have been reported (for review, see Pizzamiglio et al., 2006). Some authors, however, have reported a significant amelioration of LN signs following rehabilitation, but only on the specific tests on which LN patients were trained (e.g., Lawson, 1962; Robertson et al., 1990; Wagenaar et al., 1992). This difference, however, might be due to the short duration, frequency, and intensity of some VST protocols with respect to others. For instance, Antonucci et al. (1995) showed that VST administered for 5 days a week (8 weeks) can lead to improvements of LN signs. Most important, improvements were generalized to untrained everyday life activities.

### Limb activation treatment

Limb activation treatment consists of the joint activation of spatio-motor brain maps that enhance conscious representation of specific spatial sectors (Rizzolatti and Berti, 1990). Robertson and North (1992) (see also Robertson et al., 1992; Robertson and North, 1993) empirically tested this assumption by asking LN patients to perform voluntary movements with their contralesional hemibody. The most important finding of the first studies that investigated the effects of LAT was that a significant reduction of LN signs occurred only when two conditions were concurrently satisfied: a voluntary movement of the contralesional limb (Condition 1), performed in the contralesional space (Condition 2). The same result was observed even when a patient could not see his own moving hand (Robertson and North, 1992), suggesting that the positive effects of the left-limb movement could not be ascribed to the fact that the left limb acted as a visual cue. In fact, visual cues are known to reduce LN (Riddoch and Humphreys, 1983; Halligan et al., 1991), but they seem not to be as effective as active movements of the contralesional limb. It is also worth to mention, however, that even passive contralesional limb movements can improve LN signs (e.g., Frassinetti et al., 2001). The relevance of Robertson and North’s (1992, 1993) studies is undoubtedly remarkable. Nonetheless, the fact that only partially positive results of the application of LAT were observed in subsequent group studies (Kalra et al., 1997; Cubelli et al., 1999; Robertson et al., 2002) has raised some still unsolved questions about the effectiveness of LAT.

### Prism adaptation

Prism adaptation is a phenomenon in which the motor system adapts to new visuo-spatial coordinates imposed by prisms that “misplace” the visual stimuli along the horizontal plane (Rossetti et al., 1998). When LN patients wear prismatic goggles inducing a visual field deviation toward the right, they show a rightward error in pointing to the visual targets. When the initial part of movement is not visible, LN patients perform a motor correction toward the contralesional (left) side of space to compensate for the prism-induced error. Thus, the initial ipsilateral displacement of the visuo-motor behavior is corrected through visuo-motor adaptation. When the prismatic goggles are removed and the distal part of the arm is not visible, LN patients show a systematic contralesional (leftward) deviation of visuo-motor responses, the so-called “after-effect.” In the pioneering study by Rossetti et al. (1998) the performance of a group of LN patients was measured using standard neuropsychological tests (e.g., line bisection, line cancelation, drawing, reading), before and after a brief period of PA, with prisms inducing a 10°-rightward displacement of the visual field. Compared with a control group of LN patients exposed to goggles with neutral lenses, LN patients treated with PA showed significant improvements, which remained stable even when LN patients were tested 2 h after the end of PA. Positive and long-lasting effects of PA have been reported on both paper-and-pencil tasks and everyday life activities in a successive series of single-case and group studies (Rossetti et al., 1998; Frassinetti et al., 2002; Serino et al., 2006, 2007, 2009; Saevarsson et al., 2009; Vangkilde and Habekost, 2010; for reviews, see Luauté et al., 2006a,b; Barrett et al., 2012; Newport and Schenk, 2012; Jacquin-Courtois et al., 2013).

Some studies, however, have not confirmed the positive effects of PA. For instance, Rousseaux et al. (2006) failed to replicate the results of Rossetti et al. (1998). In a time series study, Nys et al. (2008) examined the effects of PA in LN patients who were tested within 4 weeks post-stroke. By using four treatment sessions, Nys et al. compared the PA treatment with a “placebo prism” treatment (i.e., goggles with normal, not prismatic lenses). Although PA resulted initially in faster improvements, no differences between the experimental group and the control group were found at 1-month post treatment. Note, however, that the number of treatment sessions employed by Nys et al. (i.e., four) was less than 25% of those employed by Frassinetti et al. (2002) and by Serino et al. (2007, 2009), who both used 20 sessions of PA. In addition, also Turton et al. (2010), in an RCT, did not find beneficial effects of PA. Nonetheless, the degree of the prismatic lenses used in that study (i.e., 6°) was “weaker” than that used in the studies by Frassinetti et al. (2002) and Serino et al. (2007, 2009), who both used 10° deviating, prismatic lenses. In conclusion, the number of treatment sessions and the type of lenses used, might have made the difference between studies reporting specific beneficial effects of PA and those reporting no specific effects (Nys et al., 2008) or no effects at all (Turton et al., 2010).

### Aims of the present study

According to Kerkhoff and Schenk (2012), “[…] we need empirical evidence which identifies the best treatment, the optimal amount of treatment sessions, the best combination of treatments, and provides treatment-specific predictors for therapy responders.” The present study aimed to test the effects of the three abovementioned treatments (i.e., VST, LAT, and PA), by means of a quasi-randomized clinical trial. To the best of our knowledge, the present study was the first that directly compared the effects of VST, LAT, and PA. We aimed to answer the following questions:

1) *What is the best treatment for ameliorating LN signs?* Our aim was to compare the three LN treatments (i.e., VST, LAT, and PA) to investigate which treatment could overall be the most suitable one for ameliorating LN signs.
2) *Are there differential treatment effects on specific subtypes of LN?* We wanted to investigate the possible differential treatment effects (VST, LAT, and PA) on subtypes of LN (i.e., personal, peripersonal, and extrapersonal), to find whether there could be interactions among treatments and LN subtypes.
3) *Are treatment effects observed on ecological tasks?* Bowen and Lincoln (2007) reviewed 12 RCTs regarding LN treatments. Only four RCTs had adequate allocation concealment (i.e., low risk of selection bias). Only 6 out of 12 RCTs measured disability and only 2 of them investigated whether the effects persisted. The overall effect of LN treatments on measures of disability was not statistically significant. Our aim was to investigate the effects of LN treatments on tests and tasks resembling activities of everyday life. For these reasons, in the present study we reported only outcome measures related to everyday life activities.
4) *Are treatment effects larger than those of unspecific factors?* We wanted to assess the effects of unspecific factors to differentiate their modulating role over the effects of LN treatments. The possibility of neural changes (e.g., positive spontaneous recovery and/or negative loss of neural connections) has been usually controlled by testing LN patients in the so-called “chronic phase” (e.g., about 2 or 3 months after the onset of the lesion). Nonetheless, this approach does not control appropriately the effect of neural changes because of unspecific factors (e.g., spontaneous neural reorganization, social and free-time activities, medical care, physiotherapy, environmental stimulation, etc.). In the present study, we employed a multiple baseline design with two pre-treatment assessments (i.e., A1 and A2) in order to control the role of unspecific factors affecting LN patients’ performance.
5) *Are there long-lasting treatment effects?* The efficacy and effectiveness of LN treatments depend also on the post-treatment time interval, within which positive effects of treatments can be still observed. To this aim we included two post-treatment assessments (i.e., A3 and A4) separated by a 2-week interval.

## Materials and Methods

### Participants

Thirty-three patients with right-hemisphere damage and LN were recruited. Sample numerosity was calculated *a priori*, by means of the software G∗POWER 3 (Faul et al., 2007)^1^. There were two dropouts. Thus, 31 LN patients (PA group: 11 LN patients; VST and LAT: 10 LN patients) took part in the study. All LN patients were assessed and received the rehabilitation treatments at the Neuropsychology Department of the IRCCS San Camillo Hospital (Lido-Venice, Italy).

Left neglect patients gave their written informed consent according to the Declaration of Helsinki II. Inclusion criteria comprised absence of dementia, documented both by neuropsychological history and interview, as well as by means of a neuropsychological battery involving global cognitive status [Mini Mental State Examination (MMSE); Magni et al., 1996], auditory verbal short-term memory (Digit span subtest; Orsini and Laicardi, 1997), auditory verbal long-term memory (Rey’s 15 words; Carlesimo et al., 1996), verbal fluency (Novelli et al., 1986), and verbal reasoning (Spinnler and Tognoni, 1987). Patients with documented medical history of substance abuse and psychiatric disorders were excluded from the present study. LN patients had never received LN treatments before taking part in the present study. All patients had unilateral lesions because of first stroke. Lesion sites were confirmed by Computerized Tomography (CT) or Magnetic Resonance Imaging (MRI) scans. In addition, the presence of visual field defects was evaluated by means of visual perimetry. Gender, age, education, length of illness, lesion site, and stroke type are contained in Table 1.

**Table 1:** **Demographic and neurologic data of LN patients**.

Patient ID	Treatment	Hemianopia	Gender	Education (years)	Age (years)	Lesion site	Stroke type	Time since lesion onset (days)
1	LAT	−	M	5	76	P	I	207
2	LAT	−	F	13	80	P, BN	I	40
3	LAT	+	M	17	54	TPO	H	89
4	LAT	−	M	8	39	FTP	H	95
5	LAT	−	F	17	81	LV	I	64
6	LAT	+	F	8	51	BN, IC	H	39
7	LAT	−	M	5	73	TPO	I	66
8	LAT	+	F	13	65	FTP	H	141
9	LAT	+	M	13	42	BN, IC	H	43
10	LAT	−	F	8	80	P	H	33
11	PA	−	M	8	57	T, BN	I	62
12	PA	−	F	8	75	P	I	31
13	PA	−	M	5	62	FP	H	345
14	PA	−	M	5	69	FP	I	57
15	PA	−	M	8	69	FTP	I	35
16	PA	−	F	5	59	P	I	207
17	PA	+	F	5	72	TP	I	58
18	PA	−	F	5	86	TP	I	65
19	PA	−	F	5	61	IC	H	92
20	PA	−	F	8	71	TP	I	58
21	PA	−	M	13	51	FTP	I	108
22	VST	−	M	13	70	BN	H	88
23	VST	−	M	5	86	FTP	I	132
24	VST	−	M	13	60	P, LV	I	41
25	VST	−	F	3	79	TP	I	82
26	VST	−	F	5	72	BN, LV	I	223
27	VST	−	F	8	78	FTP	I	54
28	VST	+	M	6	74	FTP	I	71
29	VST	+	M	5	57	TP, IC	H	43
30	VST	−	M	19	59	BN, IC	H	136
31	VST	+	F	13	41	TP	I	101

*F, frontal; T, temporal; P, parietal; O, occipital; BN, basal nuclei; IC, internal capsule; LV, lateral ventriculus; I, ischemic; H, hemorrhagic; +, hemianopia present; −, hemianopia absent*.

### Assessment and treatment schedule

Assessment was performed four times within a short time series. The first assessment (A1) was carried out to verify the presence and severity of LN signs. Two weeks after the end of A1, the second assessment (A2) was carried out to verify (i.e., A1 vs. A2) the effects of unspecific factors only (e.g., spontaneous neural changes, improvements to sustained attention, test–retest effects) or the effects of other therapies and activities, which were normally provided to the LN patients (e.g., pharmacological treatment, physiotherapy, social and free-time activities, environmental stimulation). Then, LN patients received the treatments for 2 weeks. The third assessment (A3) was carried out immediately after the end of the 2-week-long treatments (i.e., A2 vs. A3) to assess the effectiveness of each treatment (VST, LAT, and PA) and treatment-induced differences that were beyond and above those differences that were due only to unspecific factors (i.e., A1 vs. A2). The fourth assessment (A4) was carried out 2 weeks after the end of A3 (i.e., A3 vs. A4) to evaluate the presence of long-lasting effects of the treatments. In summary, there were two pre-treatment assessments (i.e., A1 and A2) and two post-treatment assessments (i.e., A3 and A4).

### General procedure

All right-hemisphere-damaged patients who showed LN, both on Assessments 1 and 2, on at least one subtest of the Behavioral Inattention Test (Wilson et al., 1987), the Fluff test (Cocchini et al., 2001), the Bells test (Gauthier et al., 1989), or the Room description test, were assigned to one of the treatment groups (VST, LAT, or PA), on the basis of the order of patients’ admission to the Department of Neuropsychology. That is, a quasi-randomized sequence (i.e., alternation) of the order of treatments was established. This fixed sequence was repeated in blocks (i.e., the first patient was assigned to the PA group, the second patient to the LAT group, the third patient to the VST group, the fourth patient to the PA group, and so on). All LN patients received the same neurological and neuropsychological assessments according to the rehabilitation protocol. The 2-week-long rehabilitation program consisted of 20 sessions (overall treatment duration: 2 weeks). Each session lasted approximately 20 min. There were two daily sessions (i.e., one session in the morning and one in the evening), 5 days a week.

### Visual scanning training

#### Apparatus and stimuli

Stimuli comprised black-and-white drawings. Each drawing was printed on an A4, landscape-oriented, white sheet of paper. Each drawing was divided into multiple parts. Each part had either a little black point inside or it was empty. Participants were asked to fill-out only those parts of the drawings, which had the little black point inside. The midline of each drawing was aligned with the patient’s body midline. The drawings were presented to each patient following the same order. A vertical, wide, pink-colored stripe was placed along the left edge of each sheet of paper.

#### VST procedure

Patients were required to look at the pink-colored stripe before starting to scan and fill-out each drawing. After having filled each drawing, LN patients were verbally instructed and encouraged to look again at the pink-colored stripe and, then, to check-out the drawing for possible omissions. After having checked-out for omissions, patients were presented with a new drawing and the next trial started. The verbal cue (“look at the pink-colored stripe”) remained the same through all the phases of the rehabilitation procedure; no other verbal cues were given by the examiner.

### Limb activation treatment

#### Apparatus and stimuli

This treatment involved the use of the LAT Device (LAT-D)^2^, a modified version of the original “Limb Activation Device” (LAD) employed by Robertson et al. (2002). The LAT-D comprised a central unit and a bellows. The central unit encompassed a small plastic box, measuring 11 cm × 6 cm × 3 cm (weight = 150 g). The box contained the power supply, a microcontroller, a timer, a buzzer, and a LED. The control unit could activate the buzzer and display a light, at either random or fixed intervals. The bellows (measuring 15.2 cm × 2.5 cm) could be pressed by the patients to stop a buzzing tone emitted by the buzzer. The central unit was connected with the buzzer by means of a spiral plastic air tube, so that the distance between the box and the bellows could be easily adjusted. Drawings were the same as those used for the VST procedure.

#### LAT procedure

The bellows was fixed between each patient’s left arm and the left armrest of the wheelchair. Then, LN patients were asked to fill-out the same drawings as those used in the VST. Each time LN patients heard the tone emitted by the buzzer, they were instructed to press the bellows with their left arm to turn-off the tone.

During the first week of treatment, the buzzer was set to emit the tone at a fixed time interval of 240 s, whereas in the second week of treatment the buzzer was set at a fixed time interval of 120 s. If LN patients did not move their left arm within 1 min from the onset of the tone, the examiner verbally cues reminded them to press the bellows with their left arm to turn-off the tone. No other verbal or non-verbal cues regarding the filling out of the drawings or the use of the LAT-D were given by the examiner during the task. All LN patients who completed the treatment had sufficient residual movement of the contralesional (left) arm to carry out the rehabilitation protocol.

### Prism adaptation

#### Apparatus, stimuli, and procedure

Left neglect patients were seated at a table. In front of them, a wooden box was placed on the table (height = 30 cm, width = 75 cm, depth = 34 cm at the center and 18 cm at the periphery). The box was open on the side facing the patient and on the opposite side, facing the examiner. A visual target (a pen) was presented manually by the examiner at the distal edge of the top face of the box. The visual target was presented randomly in one out of three possible positions: one central position straight ahead of the patient (0°), and two lateral positions, one on the left and one on the right of the patient’s body midline (−21 cm and +21 cm, respectively). Patients were asked to keep their right ipsilesional hand on their chest, at the level of the sternum (i.e., the hand starting position) and to point with the index finger toward the target (i.e., the pen), without hesitation. The pointing task was performed in three experimental conditions: pre-exposure (i.e., with visible and non-visible pointing), exposure (i.e., with visible pointing only), and post-exposure (i.e., with non-visible pointing only). The examiner recorded the patients’ pointing movements, as the distance between the central position of the box (0°) and the final position of the patient’s finger. A graduated scale (in cm) was used to assess the pointing deviation, which was recorded by the examiner.

The procedure was the same as that used by Frassinetti et al. (2002). All PA conditions (pre-exposure, exposure, and post-exposure) were run in each PA session.

##### Pre-exposure condition

Left neglect patients were required to point with their right index finger toward 30 targets, randomly presented at one of the three possible positions (10 targets in the center, 10 on the right, 10 on the left), with visible pointing (only first and eleventh session). Note that in visible pointing, the arm movement was performed below the top face of the box, but the index finger was visible at the final stage of pointing. Afterward, LN patients were required to point with their right index finger toward 30 new targets, which were again randomly presented at one of the three possible positions (10 targets in the center, 10 on the right, 10 on the left). The pointing movement was now performed entirely below the top face of the box, so that the index finger was not visible at any stage (i.e., non-visible pointing).

##### Exposure condition

Left neglect patients performed the same task wearing the prismatic goggles^3^. The goggles were fitted with wide-field prismatic lenses inducing a 10° shift of the visual field to the right. Patients were asked to point with their right index, without hesitation, to 90 targets presented in a random order in each of the three possible positions (30 targets in the center, 30 on the right, and 30 on the left). During the exposure condition, the arm movement was hidden below the top face of the box, except for the final part of the movement, where the index finger could emerge beyond the distal edge of the top face of the box to permit patients to see their finger.

##### Post-exposure condition

Immediately after removal of the prisms, LN patients were required to point toward 30 targets (10 in the center, 10 on the right, and 10 on the left). The pointing movement was performed entirely below the top face of the box, so that the index finger was not visible at any stage (i.e., non-visible pointing).

### Outcome measures

#### Tests for assessing personal LN

##### Comb and razor test

This test was based on Beschin and Robertson (1997) test, but we used a more sensitive formula to quantify LN patients’ performance (McIntosh et al., 2000). The equipments consisted of a comb, a razor with shield on, and a powder compact. The examiner sat opposite to the patient and held up the comb, while saying: “I would like you to show me how this comb can be used.” In the razor condition, which was used with men, the patient was told: “I would like you to show me how this razor can be used.” In the powder compact case, which was applied to women, the patient was told: “I would like you to show me how this powder compact case can be used.”

Left neglect patients were required to perform each task for 30 s. Each task was videotaped. The number of strokes on each task was analyzed off-line, by two examiners. Finally, each stroke was classified into three categories (left-sided, right-sided, or ambiguous).

The modified formula that we used to calculate the lateral bias of LN patients’ behavior was:

$$\%bias=\frac{right-left\;strokes}{left+ambiguous+right\;strokes}\times 100$$

Rightward bias yielded a positive percentage score, whereas leftward bias yielded a negative percentage score (cut-off: % bias > 11).

##### Fluff test

This test encompassed 24 targets (i.e., round felt pads; diameter = 2 cm) (Cocchini et al., 2001). Each felt pad was self-adhesive to be easily attached to the patients’ clothes, by using only little pressure. There were three targets on the right and three on the left of the trunk’s midline, six targets along the patient’s left arm, six along the right leg, and six along the left leg. No targets were placed on the right arm, because LN patients performed the task by using that arm. Each patient was blindfolded and seated, while the targets were attached. Patients were not told how many targets were attached. While the examiner attached each target, patients were distracted by engaging them in a conversation to prevent them from counting the targets. When the examiner finished attaching the targets, patients were asked to remove them, while the patients were still blindfolded. There was no time limit for the response and the test finished when the patients declared that they had collected all the targets. Only target omissions on the left were considered for determining the cut-off score, which was<13 out of 15.

#### Tests for assessing peripersonal LN

##### Picture scanning

In this test three large photographs were presented to the patients, one at a time (Wilson et al., 1987). The photographs depicted: a meal, a wash basin and toiletries, and a large hospital room containing various pieces of furniture and hospital aids. The midline of each photograph was aligned with the body midline of each patient. The patients were instructed to name and/or point to the items in each photograph. The number of identified targets was scored. There was no time limit for the patients to perform the test. The cut-off score of this subtest was ≤5 identified targets out of 9.

##### Menu reading

This task consisted of an “open-out” page containing 24 common words of food items arranged in four adjacent columns (two on the left page and two on the right page) (Wilson et al., 1987). Patients were asked to read aloud out all the words. Responses on each of the 24 words were scored as correct or incorrect. Incorrect responses consisted of partial/whole word substitutions or omissions. There was no time limit for the patients to perform the test. The cut-off score of this subtest was ≤8 correct responses out of 9.

##### Coin sorting

In this test the patient had to indicate coins of different values, as requested by the examiner (Wilson et al., 1987). Coins were distributed to the left, to the right, and in front of the patients, according to a standard arrangement scheme on a board. The midline of the board was aligned with the body midline of each patient. There were 3 coins for each value, for a total of 15 coins. The examiner recorded the indicated coins. There was no time limit for the patients to perform the test. The cut-off score of this subtest was ≤8 indicated coins out of 9.

##### Semi-structured ecological scale

This scale was developed to assess the qualitative/quantitative asymmetries present in the exploration of space in LN patients, in situations similar to those of everyday life (Zoccolotti and Judica, 1991). In the present study, we used only the subtests A (Serving tea) and C (Card dealing). During these subtests, patients sat at a table. They were required to take from the table and distribute the tea/the cards to three examiners, who were seated around the table (one examiner on the left, one on the right, and one in front of the patient). Patients’ performance on these subtests was videotaped. Then two examiners evaluated off-line the patients’ performance, according to the scoring system provided with the test. Scoring was based on a four-level scale, which evaluated how accurately LN patients served the tea or distributed the cards. The maximum score was 0 (i.e., no neglect), whereas the minimum score was 3 (i.e., severe neglect). There was no time limit for the patients to perform the test.

#### Test for assessing extrapersonal LN

We assessed the performance of LN patients in the extrapersonal space. There are not yet standardized measures of LN for the extrapersonal space, defined as the locomotor space beyond the reaching and grasping space. For this reason we tested LN patients in a room (7 m × 4 m), which was provided with various objects and pieces of furniture arranged symmetrically with respect to the room’s midline (10 targets on the left and 10 targets on the right; maximum score: 20). LN patients sat on their wheelchair at the center of one of the two 10-meter-long walls of the room. Then, they were asked to describe all the targets that they could see. The examiner, standing behind each patient, recorded their responses on a map of the room depicting the positions of all the targets. There was no time limit for the patients to perform the test.

#### The Catherine Bergego Scale

We also assessed the presence and degree of LN in everyday life situations (Azouvi et al., 2006). To this aim we used the standardized 10-item checklist provided with the CBS. Each item of the CBS was responded on a four-point rating scale (range: 0 = “no LN-related difficulties”; 4 = “presence of severe LN-related difficulties”). In the present study the CBS was administered as a questionnaire to the patients’ caregivers.

## Results

Left neglect patients in the three treatment groups did not differ for age, education, time since lesion onset, and on the MMSE (all *p*s *ns*). Only the performance of the patients with complete data on all four Assessments (i.e., 31) was subjected to the statistical analyses. Two-way, mixed ANOVAs were run, with Intervention type (VST, LAT, and PA) as the between-participants factor and Assessment (A1, A2, A3, and A4) as the within-participants factor. Wherever sphericity was violated, Huynh–Feldt corrections were applied.

### Personal space

#### Fluff test

**Figure 1:**
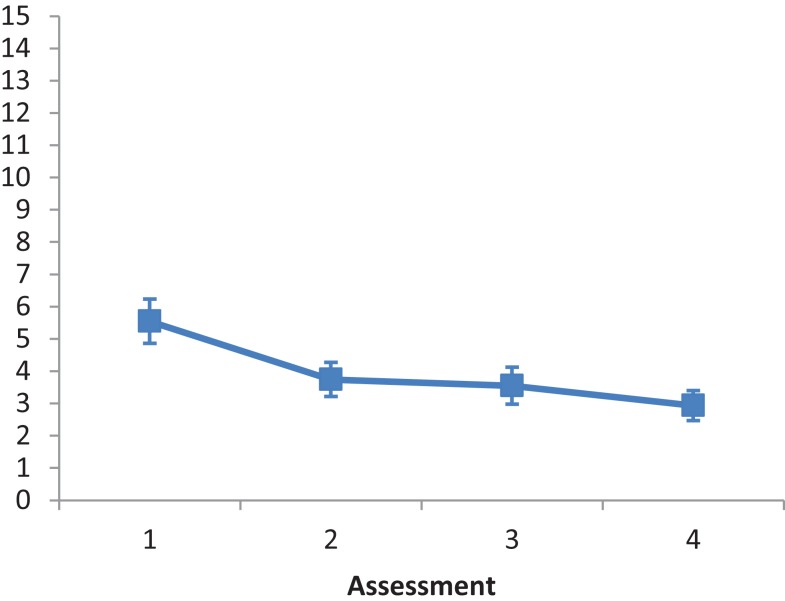
**LN patients’ performance on the Fluff test as a function of assessment**. Error bars represent 1 SEM.

The main effect of Intervention type was not significant, *F*(2, 28) = 1.015, *ns*. The main effect of Assessment was significant, *F*(3, 84) = 5.187, *p* < 0.001, partial eta squared = 0.156. A repeated contrast showed that only the difference between Assessment 1 and Assessment 2 was significant, *F*(1, 28) = 5.848, *p* < 0.05, partial eta squared = 0.173 (see Figure 1). The interaction Intervention type by Assessment was not significant, *F*(6, 84) = 0.835, *ns*.

#### Comb and razor test

The main effect of Intervention type was not significant, *F*(2, 29) = 0.149, *ns*. The main effect of Assessment was not significant, *F*(3, 87) = 1.428, *ns*. A repeated contrast revealed no significant differences among the four levels of Assessment (all *p*s *ns*). The interaction Intervention type by Assessment was not significant, *F*(6, 87) = 1.173, *ns*.

### Peripersonal space

#### Picture scanning subtest

**Figure 2:**
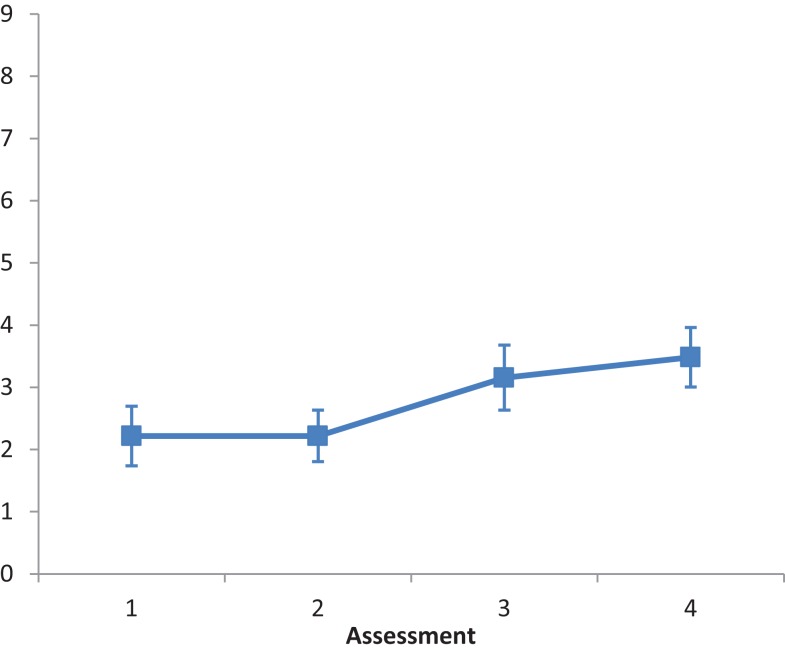
**LN patients’ performance on the Picture Scanning subtest as a function of assessment**. Error bars represent 1 SEM.

The main effect of Intervention type was not significant, *F*(2, 28) = 3.088, *ns*. The main effect of Assessment was significant, *F*(2.647, 74.112) = 7.414, *p* < 0.001, partial eta squared = 0.209. A repeated contrast showed that only the difference between Assessment 2 and Assessment 3 was significant, *F*(1, 28) = 7.003, *p* < 0.05, partial eta squared = 0.2 (see Figure 2). The interaction Intervention type by Assessment was not significant, *F*(5.294, 74.112) = 1.260, *ns*.

#### Menu reading subtest

**Figure 3:**
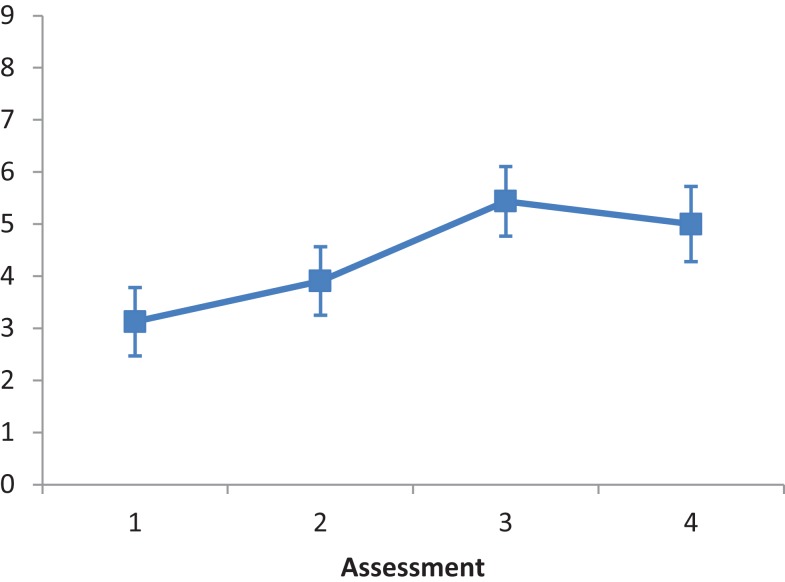
**LN patients’ performance on the Menu Reading subtest as a function of assessment**. Error bars represent 1 SEM.

The main effect of Intervention type was not significant *F*(2, 28) = 1.542, *ns*. The main effect of Assessment was significant, *F*(3, 84) = 8.849, *p* < 0.001, partial eta squared = 0.233. A repeated contrast showed that only the difference between Assessment 2 and Assessment 3 was significant, *F*(1, 28) = 7.582, *p* < 0.05, partial eta squared = 0.213 (see Figure 3). The interaction Intervention type by Assessment was not significant, *F*(6, 84) = 0.488, *ns*.

#### Coin sorting subtest

The main effect of Intervention type was not significant *F*(2, 28) = 2.323, *ns*. The main effect of Assessment was not significant, *F*(3, 84) = 2.390, *ns*. A repeated contrast revealed no significant differences among the four levels of Assessment (all *p*s *ns*). The interaction Intervention type by Assessment was not significant, *F*(6, 84) = 1.487, *ns*.

#### Semi-structured ecological scale

##### Subtest A (serving tea)

**Figure 4:**
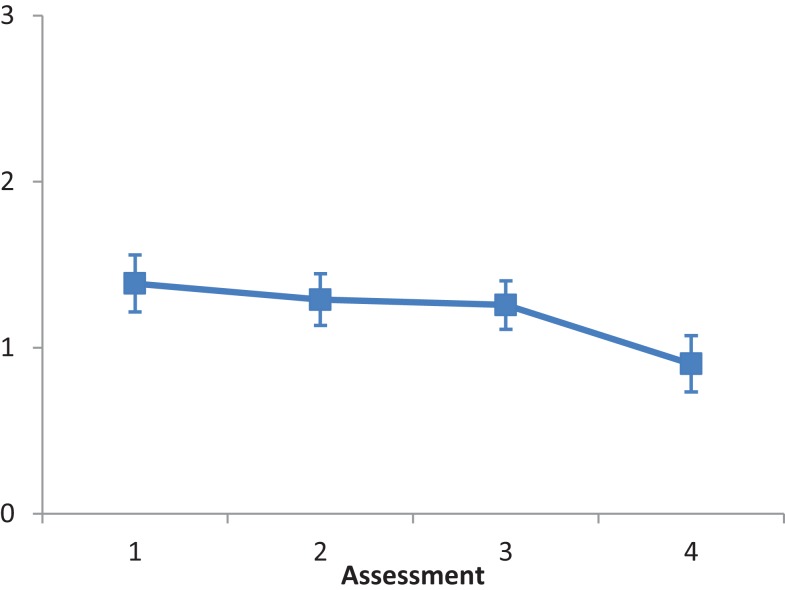
**LN patients’ performance on the Serving tea subtest as a function of assessment**. Error bars represent 1 SEM.

The main effect of Intervention type was not significant, *F*(2, 28) = 1.819, *ns*. The main effect of Assessment was significant, *F*(3, 84) = 3.862, *p* < 0.001, partial eta squared = 0.121. A repeated contrast showed that only the difference between Assessment 3 and Assessment 4 was significant, *F*(1, 28) = 7.81, *p* < 0.05, partial eta squared = 0.218 (see Figure 4). The interaction Intervention type by Assessment was not significant, *F*(6, 84) = 1.972, *ns*.

##### Subtest C (card dealing)

**Figure 5:**
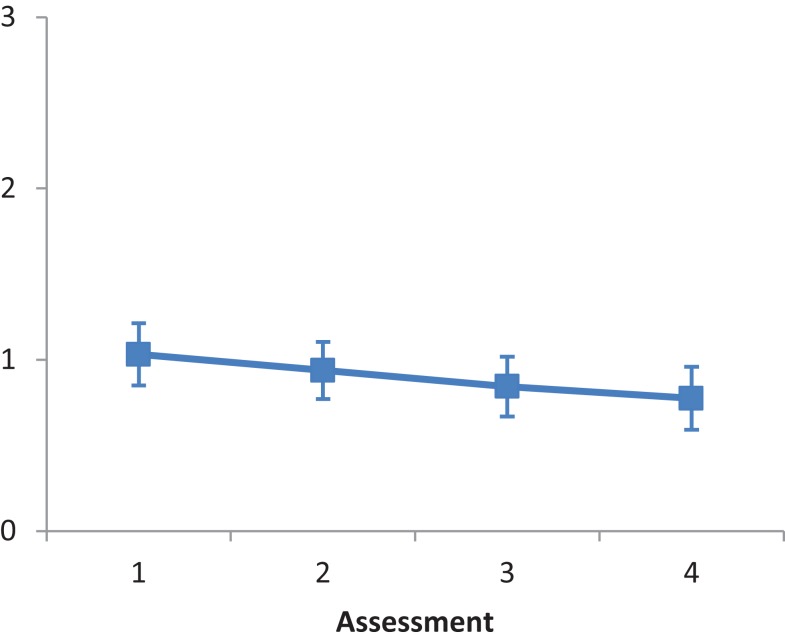
**LN patients’ performance on the Card dealing subtest as a function of assessment**. Error bars represent 1 SEM.

The main effect of Intervention type was not significant, *F*(2, 28) = 0.260, *ns*. The main effect of Assessment was significant, *F*(1.271, 35.583) = 32.947, *p* < 0.001, partial eta squared = 0.541. A repeated contrast showed that the differences between Assessment 2 and Assessment 3, and between Assessment 3 and Assessment 4 were significant: *F*(1, 28) = 35.254, *p* < 0.05, partial eta squared = 0.557, and *F*(1, 28) = 35.637, *p* < 0.05, partial eta squared = 0.560, respectively (see Figure 5). The interaction Intervention type by Assessment was not significant, *F*(2.542, 35.583) = 1.874, *ns*.

### Extrapersonal space

#### Room description

The main effect of Intervention type was not significant, *F*(2, 28) = 0.436, *ns*. The main effect of Assessment was not significant, *F*(3, 84) = 1.093, *ns*. A repeated contrast revealed no significant differences among the four levels of Assessment (all *p*s *ns*). The interaction Intervention type by Assessment was not significant, *F*(6, 84) = 0.581, *ns*.

#### CBS

**Figure 6:**
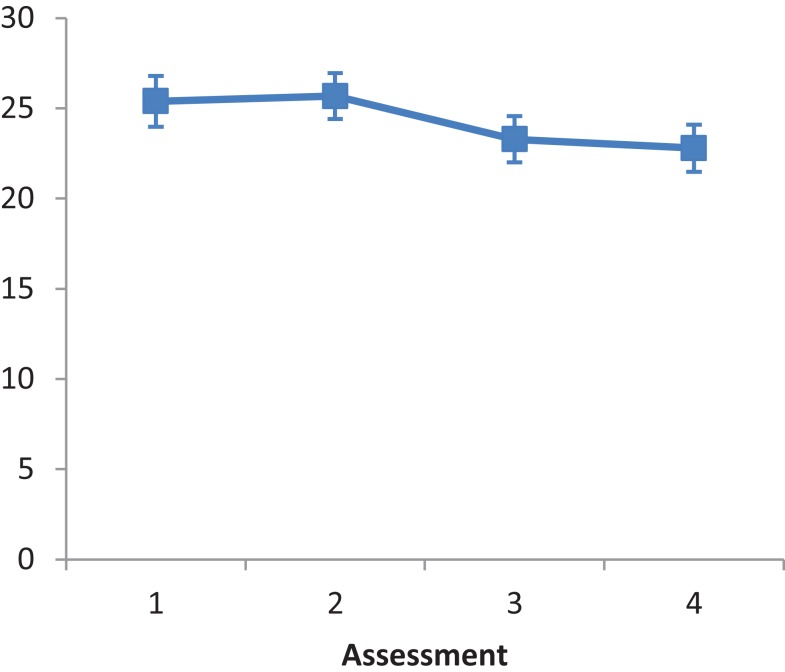
**LN patients’ performance on the CBS as a function of assessment**. Error bars represent 1 SEM.

The main effect of Intervention type was not significant, *F*(2, 25) = 0.274, *ns*. The main effect of Assessment was not significant, *F*(2.196, 54.9) = 2.615, *ns*. A repeated contrast showed that the differences between Assessment 2 and Assessment 3 was significant, *F*(1, 25) = 5.489, *p* < 0.05, partial eta squared = 0.180 (see Figure 6). The interaction Intervention type by Assessment was not significant, *F*(4.392, 54.9) = 0.220, *ns*.

## Discussion

In the present study we compared, for the first time, the overall and differential effects of three of the most widely used LN treatments: VST, LAT, and PA. LN patients’ performance was assessed four times: A1 and A2 (i.e., the two pre-treatment assessments); A3 and A4 (i.e., the two post-treatment assessments). LN patients were treated for the same number of sessions (i.e., 20). Our aims were to:

1. Test the overall efficacy and effectiveness of VST, LAT, and PA.
2. Test the differential effects of VST, LAT, and PA on specific subtypes of LN (e.g., personal, peripersonal, and extrapersonal).
3. Test the effects of VST, LAT, and PA on measures of everyday life activities.
4. Test the specific effects of LN treatments (A2 vs. A3) above and over the effects of unspecific factors (A1 vs. A2).
5. Test the long-lasting effects of LN treatments (A3 vs. A4).

In the following paragraphs each of our aims is discussed in relation to our findings.

1. We compared for the first time VST, LAT, and PA. In recent reviews, both PA and LAT, as well as VST have been proposed as the gold standard of LN rehabilitation: LAT and VST (Riestra and Barrett, 2013), PA (Mattingley, 2002). We found a main effect of treatments, but we did not find significant interactions between treatments and assessment sessions. That is, it seems that all three treatments can lead to similar positive outcomes concerning LN rehabilitation. Apparently VST, LAT, and PA are based on different principles of functioning. PA is thought to recalibrate ipsilesionally biased proprioceptive and visuo-spatial coordinates, LAT presumably activates joined spatio-motor representations of the contralesional space, and VST leads to compensatory, voluntary, contralesional scanning. To explain, however, the absence of differences in the present study, it can be assumed that beyond the supposed differences, VST and LAT activate some kind of voluntary orienting of spatial attention toward the contralesional space. Indeed, during both VST and LAT, LN patients are required to perform voluntary actions within (i.e., LAT) or toward the contralesional space (i.e., VST). This, in turn, may lead to the re-allocation of residual spatial resources toward the contralesional space. In contrast, PA does not activate some kind of voluntary orienting of spatial attention: left after-effect observed after removing the prismatic goggles is induced by automatic processes during the PA procedure.
  A working hypothesis can be that LN can have different underlying causes, each addressed by a different kind of treatment. If this is the case, then additive effects of LAT, PA, and VST should be observed. The additive effects of treatments can be addressed in future studies in which the combined use of the three treatments should be tested (e.g., LAT or PA vs. LAT plus PA; for reviews on additive effects of LN treatments, see Singh-Curry and Husain, 2008; Saevarsson et al., 2011). For instance, by combining neck vibration and PA, Saevarsson et al. (2010) have reported additive therapeutic effects on LN signs. Nonetheless, some studies have reported no better effects of combined treatments with respect to single treatments for LN (e.g., Pizzamiglio et al., 2004; Keller et al., 2009). Thus, further studies are required to explore the presence of possible additive effects of LN treatments and/or propose a global approach to the rehabilitation of LN patients.
2. We found different effects of treatments in relation to LN subtypes. That is, the effects of VST, LAT, and PA were present only on tests assessing the peripersonal space (i.e., the within-reaching space). Instead, we did not find any effects of LAT, PA, or VST on tests tapping the personal (i.e., the body space) or the extrapersonal (i.e., the locomotor space). We think that this finding is not surprising given that, in all three treatments, LN patients were required to perform actions only within their peripersonal space. Our findings are in accordance with Pizzamiglio et al. (1992). In contrast, our findings are partially different from those of Frassinetti et al. (2002) and Serino et al. (2007), who found positive effects of PA not only for the peripersonal space but also for the personal and the extrapersonal space. With reference to the peripersonal space, however, Frassinetti et al. (2002) and Serino et al. (2007) used totally or partially different procedures in administering the Fluff test (i.e., LN patients were not blindfolded while searching for the targets), whereas we used the standard procedure (i.e., patients were always blindfolded; Cocchini et al., 2001). In addition, neither Frassinetti et al. (2002) nor Serino et al. (2007) used the Comb and Razor test. Regarding the exploration of the extrapersonal space both Frassinetti et al. (2002) and Serino et al. (2007) tested their patients in a rather small room (3.6 m × 2.2 m), whereas we used a considerably larger room (7 m × 4 m). These procedural differences should be addressed in future studies. We propose that a possible way for extending the positive effects of LAT, PA, and VST, found in the peripersonal space, can be that of including versions of the three treatments, in which LN patients are required to perform actions not only in the peripersonal but also in the personal and the extrapersonal spaces. Note that some generalization to untreated tasks has been reported with reference to PA (e.g., reading, wheel-chair driving, auditory extinction, representational neglect, mental imagery; for review, see Jacquin-Courtois et al., 2013). Nonetheless, the exact extend of personal, peripersonal, and extrapersonal aspects in these tasks is unclear.
3. One of the major critiques regarding LN rehabilitation (Bowen and Lincoln, 2007) is that previously reported positive findings have used outcome measures of impairment (e.g., paper-and-pencil tests such as cancelation tests, drawing tasks, or line bisection), but not measures concerning disability (e.g., tasks resembling or directly investigating activities of everyday life). We tested the effects of LAT, PA, and VST on everyday life activities (Bergego questionnaire) and on tasks resembling everyday life activities (e.g., looking at photographs, reading, etc.). We found that positive outcomes were observed as a consequence of LN treatment. Our findings are in accordance and further extend the findings of previous single-case and group studies in which positive effects of VST, LAT, and PA on LN patients have been reported (e.g., Antonucci et al., 1995; Kalra et al., 1997; Frassinetti et al., 2002). We think, thus, that our study adds one more step toward accepting the efficacy and effectiveness of LAT, PA, and VST in the rehabilitation of LN. Note, however, that we did not find positive results of treatments on some tests, namely Coin sorting and Serving tea. A possible reason for these negative findings might be that both tests are the only ones in which patients are required to reach out to touch (Coin sorting) and reach out to grasp (Serving tea) real objects in the peripersonal space. Thus, this might be a case of task-specific effects of treatments, given that in none of our treatments the patients were required to interact with real objects. Nonetheless, instead of the requirement on reaching out and grasping, it could be that the “Coin sorting” and “Serving the tea” tests are just not very sensitive tasks for revealing treatment-associated changes in LN. In future investigations, the treatments used in the present study might be modified to include some interaction with real objects within more sensitive tests.
4. One might attribute the reported main effects of our treatments to unspecific factors (e.g., spontaneous neural reorganization, social and free-time activities, medical care, physiotherapy, environmental stimulation, test-retest effects, global improvements in sustained attention, etc.). We do not think that this is the case for the following reasons. First, there is no reason why an unspecific effect of treatments should be observed only in the specific sector of space (i.e., the peripersonal space), which was the target space of all actions performed by LN patients tested. Second, the effects of unspecific factors cannot account for the absence of positive results regarding tasks performed in the peripersonal space that required reaching out or grasping of real objects (i.e., non-treated actions). Third, we controlled methodologically and statistically for the effects of unspecific factors only, by employing two pre-treatment assessments (A1 and A2), which were spaced by a 2-weeks interval. The results showed that there were no differences between the patients’ performance on Assessment 1 and Assessment 2, for those measures where, instead, a significant difference between patients’ performance on Assessment 2 and Assessment 3 was observed. Fourth, the danger that our effects were due only to unspecific factors was further controlled in the comparison between LN patients’ performance on Assessment 1 and 2. In that time interval, all patients received daily sessions of physiotherapy. Patients are usually highly motivated to participate in physiotherapy sessions, given that motor defects are more obvious to the patients, than LN-related defects. During physiotherapy sessions, the patients were provided with unstructured cues to attend to the left, while the physiotherapist is placed for most of the time to the left of the patients’ body midline. Thus, if our effects were due to simply “doing something” or to motivational factors, beneficial effects would have been observed in most comparisons between Assessment 1 and 2. By contrast, this was not the case. Finally, we reasoned that unspecific effects – not related to treatments – would have ameliorated LN patients’ performance not only on spatial but also on non-spatial tasks. To this aim, we ran repeated contrasts, on the Intervention type factor, on three non-spatial tests: verbal reasoning, semantic verbal fluency, and digit span. The results showed that none of these contrasts was significant (i.e., A1 vs. A2; A2 vs. A3; A3 vs. A4). Instead, in our time series only the introduction of treatment leaded to improvements in the abovementioned comparisons. To the best of our knowledge this is the first study reporting specific effects of LAT and PA compared with the effects of unspecific factors (for evidence regarding VST, see Pizzamiglio et al., 1992; Paolucci et al., 1996).
  Another possible critical point of our study is that we did not employ a “typical” “control” group. Note, however, that we have employed VST. In most of the previous studies on LN rehabilitation, VST has been employed as a control treatment (for review, see Riestra and Barrett, 2013). The VST, however, is one rehabilitation treatment (i.e., not a “doing nothing” or unspecific treatment). For this reason we did not name VST, in our study, a control treatment, but we considered it as an alternative treatment. We think that this is the most appropriate term (i.e., alternative treatment) to use when referring to VST. Each of the three treatments (LAT, PA, and VST) has been extensively compared with different control treatments (see Riestra and Barrett, 2013). Thus, it is thought that each of these treatments can be considered as a valid treatment for rehabilitating LN. Nonetheless, to date, no study has compared the differential effectiveness and efficacy of these three treatments. We conducted, indeed, the present study to test which would be the best LN treatment and which LN treatment would have worked better with specific LN subtypes. We considered that, in turn, each treatment would be compared with the two other treatments. In this sense, in the present study we had, for each comparison, not only one but two control treatments (LAT vs. PA/VST; PA vs. LAT/VST; VST vs. PA/LAT). Adding, for example, a non-treatment group would have been problematic for ethical reasons (see also discussion on the possible effects of unspecific factors).
5. An important point regarding LN rehabilitation is the stability of positive effects in time. Indeed, the efficacy and effectiveness of LN treatments is also based on the time interval during which positive effects of LN can be maintained. In the present study we showed that positive effects of treatments can be maintained for at least 2 weeks following the end of each treatment; further improvement was observed in one measure (i.e., Card dealing). Note, however, that on the Serving tea subtest we observed LN improvement only in the comparison between A3 and A4. A possible explanation is that beneficial effects of treatments on this test require more time to be consolidated. Further studies employing this test are required to clarify this point. Our findings are in accordance with those of Frassinetti et al. (2002) and Serino et al. (2007) with reference to PA, and with the findings of Pizzamiglio et al. (1992) with reference to VST. To the best of our knowledge our group study is the first one reporting long-lasting effects also of LAT on measures of LN in everyday life.

In summary, although we used only a small number of treatment sessions (20 sessions over a 2-week interval), an amelioration of LN signs was observed in the majority of the ecological tests assessing the peripersonal space and in everyday life activities measured with the CBS. Our findings cannot be attributed to unspecific factors, and lasted for at least 2 weeks after the end of each treatment. Further studies, however, are required to better investigate which is the most effective rehabilitation procedure for improving processing of the personal and the extrapersonal space, presumably by adapting existing treatment procedures. We employed standardized and rather varying tests for performing LN assessment. These tests are considered the “gold standard” for exploring and investigating different LN subtypes. On the basis of our findings we cannot advance any recommendation regarding the sensitivity of each of the tests that we used. Given that some dissociations were observed among tests of peripersonal space (and between tests of personal, peripersonal, and extrapersonal space) we recommend that comprehensive batteries, instead of single tests, be used to assess different LN subtypes.

Some authors have suggested that PA (Mattingley, 2002; Luauté et al., 2006a) or LAT and VST (Riestra and Barrett, 2013) might each be the best LN treatment. Nonetheless, these treatments had never been directly compared in previous studies. We suggest, instead, that all three treatments can be considered as valid rehabilitation interventions and should be employed for ameliorating LN signs.

## Conflict of Interest Statement

The authors declare that the research was conducted in the absence of any commercial or financial relationships that could be construed as a potential conflict of interest.

## Footnotes

^1^http://www.psycho.uni-duesseldorf.de/abteilungen/aap/gpower3/

^2^http://www.treatneglect.co.uk/prod01.htm

^3^http://www.optiquepeter.com/en/index.php
